# Opioid use disorder in two samples of the Lebanese population: scale validation and correlation with sleep and mood disorders

**DOI:** 10.1186/s12888-023-05304-8

**Published:** 2023-11-01

**Authors:** Karam Chamoun, Joseph Mouawad, Pascale Salameh, Hala Sacre, Ramzi Haddad, Lydia Rabbaa Khabbaz, Bruno Megarbane, Aline Hajj

**Affiliations:** 1 Faculty of Pharmacy, Saint Joseph University of Beirut, Beirut, Lebanon; 2 Laboratoire de Pharmacologie, Pharmacie Clinique et Contrôle de Qualité des Médicaments, Saint Joseph University of Beirut, Beirut, Lebanon; 3 Inserm, UMR-S1144, Université Paris Cité, Paris, France; 4 INSPECT-LB (Institut National de Santé Publique, d’Épidémiologie Clinique et de Toxicologie- Liban), Beirut, Lebanon; 5 School of Medicine, Lebanese American University, Byblos, Lebanon; 6 Faculty of Pharmacy, Lebanese University, Hadat, Lebanon; 7 Department of Primary Care and Population Health, University of Nicosia Medical School, Egkomi, Nicosia 2417 Cyprus; 8 Psychiatry Department, Faculty of Medical Sciences, Lebanese University, Beirut, Lebanon; 9 Psychiatry Department, Faculty of Medicine, Saint Joseph University, Beirut, Lebanon; 10 Department of Medical and Toxicological Critical Care, Federation of Toxicology, Lariboisière-Fernand Widal Hospital, Paris, France; 11 Faculté de Pharmacie, Université Laval, Québec city, Québec Canada; 12 Oncology Division, CHU de Québec- Université Laval Research Center, Québec City, Québec Canada

**Keywords:** Arabic, Lebanon, Opioid risk Tool, Opioid use disorder, Sleep disorders, Scale validation

## Abstract

**Background:**

The revised Opioid Risk Tool (ORT-OUD) is a brief, self-report scale designed to provide clinicians with a simple, validated method to screen for the risk of developing an Opioid Use Disorder (OUD) in patients without a prior history of substance abuse. This study aimed to translate and validate the Arabic version of ORT-OUD in the Lebanese population and assess its clinical validity in a sample of patients with OUD.

**Methods:**

This cross-sectional study in the Lebanese population used several validated scales to assess the risk of OUD, including the Alcohol, Smoking, and Substance Involvement Screening Test (ASSIST). Other tools evaluated chronotype and sleep and mood disturbances. Principal component analysis with Varimax rotation was applied to assess ORT-OUD construct validity. Convergent validity with the Arabic version of ASSIST was evaluated. The ORT-OUD criterion validity was then assessed in a clinical sample of patients with OUD.

**Results:**

This study included 581 participants. The prevalence of the OUD risk in the Lebanese population using the ORT-OUD scale and the ASSIST-opioids scale was estimated at 14.5% and 6.54%, respectively. No items of the ORT-OUD were removed; all items converged over a solution of four factors with an eigenvalue > 1, explaining a total of 68.2% of the variance (Cronbach’s alpha = 0.648). The correlation coefficients between the ORT-OUD total score and ASSIST subscales were as follows: ASSIST-opioids (r = 0.174; p = < 0.001), ASSIST-sedatives (r = 0.249; p < 0.001), and ASSIST-alcohol (r = 0.161; p = < 0.001). ORT-OUD clinical validation showed a correlation with ASSIST-opioids (r = 0.251; p = 0.093) and ASSIST-sedatives (r = 0.598; p < 0.001). Higher ORT-OUD scores were associated with a family and personal history of alcohol and substance consumption and higher insomnia and anxiety scores.

**Conclusions:**

This study is the first to validate the Arabic version of ORT-OUD in the Lebanese population, an essential step towards improving the detection and management of OUD in this population.

**Supplementary Information:**

The online version contains supplementary material available at 10.1186/s12888-023-05304-8.

## Background

The opioid crisis is considered a global health issue [1]. It originated in the 1990s with the surge in opioid analgesics prescribing, particularly oxycodone, resulting in overdoses and fatalities attributed to their use; these have steadily increased ever since [2]. A second wave of deaths due to heroin occurred in 2010. It is estimated that 80% of heroin users in the United States initiated their substance use with prescription opioids [3]. Finally, a third wave of deaths emerged in 2013, primarily attributed to synthetic opioids, notably fentanyl and its analogs [4]. The opioid crisis was declared a national public health emergency on October 27, 2017. Opioids accounted for nearly 75% of all overdose deaths in 2020 [5]. By June 2021, synthetic opioids were involved in approximately 87% of opioid-related deaths and 65% of all overdose deaths [6]. As of today, over 108,000 overdose deaths occurred during the 12-month period ending in April 2022, according to data from the Centers for Disease Control and Prevention (CDC) [6]. In the United States alone, more than 250 million opioid prescriptions are recorded annually, with an exponential increase in prescriptions over the past fifteen years, accompanied by a rise in the number of hospitalizations and deaths caused by opioid overdose [7]. Moreover, the increased accessibility of opioids has led to massive medication hoarding and diverted consumption for nonmedical purposes [8], contributing to persistent issues such as Opioid Use Disorder (OUD).

In Lebanon, data on opioid consumption are scarce. However, there has been longstanding evidence of nonmedical use of prescription drugs, and recent years have witnessed a significant surge in opioid and psychoactive substance use [9, 10]. The Lebanese Ministry of Public Health [11] reported a six-fold increase in opioid prescriptions from 1995 to 2001. Furthermore, Decree number 1/480 simplified the prescription process by only requiring a basic medical report describing the disease and the cause of pain for non-cancer patients [12]. According to the 2011 Global School Health Survey (GSHS), the prevalence of illicit substance and/or prescription drug use among students aged 13–15 in Lebanon was found to be 5%, compared to 3.5% in 2005, with a growing concern over the high utilization of nonprescription pharmaceutical opioids. Prescription opioids seem to be readily available, as almost two-thirds (63.4%) of university students reported ease in obtaining opioids without a prescription [10, 13]. Moreover, Lebanon has experienced numerous conflicts, political turmoil, and monetary instability since 1975, further aggravated by the recent economic collapse and the COVID-19 pandemic [14]. Since October 2019, the country has been grappling with overlapping crises, political unrest, sporadic violence, uncertainty about the future, and a lingering sense of insecurity. The massive blast at the port of Beirut on August 4, 2020, further weakened the Lebanese population, making it vulnerable to mental disorders, including anxiety, sleep disorders, and post-traumatic stress disorder [15]. This climate has likely increased stress and distress levels among the Lebanese population, precipitating mental health and sleep problems that may contribute to medication abuse and use disorders, both in prescription and illegal drugs [16]. More importantly, a study revealed variability in the frequency of follow-up by Lebanese practitioners regarding opioid prescriptions, with a substantial proportion of these practitioners not assessing additional risk factors before prescribing opioids to patients, which may further contribute to increasing OUD cases [17].

OUD stands as the most lethal consequence of opioid use and is at the core of the opioid crisis. It can lead to several social and economic harmful repercussions [18], affecting various aspects of the quality of life, including physical health, psychological health, social relationships, and environment [19]. OUD has also been associated with increased hospital admissions, emergency department visits, and risk of premature mortality, with a staggering 200% rise in overdose deaths from 2000 to 2014 [20]. Consequently, prioritizing primary prevention strategies becomes crucial, focusing on identifying risks through scalable prevention services and techniques.

Identifying patients at risk of OUD before initiating chronic opioid therapy is crucial in preventing abuse and implementing sustainable prescription drug monitoring programs. Rating scales play a considerable role in this process by helping identify individuals at higher risk of developing OUD. Hence, several scales have been developed to detect substance use-related health risks and substance use disorders [21–25]. Some of these scales are designed to screen for multiple substances, including opioids, in adults already taking opioid medications for pain management. Examples of such scales include the Current Opioid Misuse Measure (COMM^®^) [22], the Patient Medication Questionnaire (PMQ) [23], and the Alcohol, Smoking, and Substance Involvement Screening Test (ASSIST) [24]. Other tools, such as the Diagnosis, Intractability, Risk, Efficacy (DIRE) scale [26], the Screener and Opioid Assessment for Patients with Pain-Revised (SOAPP^®^-R) [25], and the Opioid Risk Tool (ORT) [21], focus on predicting aberrant drug use disorder before the initiation of long-term opioid therapy. However, most of these tools comprise 17 to 24 items, thus requiring a considerable amount of time to complete and calculate the total risk score during evaluation, except for the revised version of ORT (ORT-OUD). ORT-OUD comprises only nine items and has demonstrated superior predictive ability for OUDs [27]. Its specificity for opioids and availability in smartphone applications, such as MDCalc^®^, makes it convenient for screening OUD risk with increased bedside accessibility [28]. While ORT-OUD has been validated in various languages, it has not been validated in Arabic [21, 29], and no study in Lebanon has yet evaluated the risk of developing an OUD after opioid treatment or its correlation with other disorders.

This study aimed to translate and validate the Arabic version of the ORT-OUD in the Lebanese population and evaluate its clinical validity in a sample of patients with OUD. The secondary objective was to assess the correlates of the risk scores with sociodemographic and clinical factors, including sleep disorders, chronotype, anxiety, and depression.

## Methods

### Study design

#### General population (sample 1)

As a first step, a cross-sectional study was conducted between November 2021 and January 2022 using an online questionnaire created on Google Forms in English and Arabic (English: https://forms.gle/wT8mTFJpKsbK4A2M9; Arabic: https://forms.gle/Jxtrpzyp8hjzutth6). Snowball sampling was applied to recruit the sample. The survey was shared on social media platforms because of pandemic-related restrictive measures and to ensure better access to all the Lebanese regions to enhance the representativeness of the sample. All Lebanese adults (over 18) with access to the Internet were eligible to participate (no incentive was offered in return for their participation). A total of 581 respondents from the general population filled out the questionnaire, which required around 20 min to complete.

To assess test-retest reliability, the ORT-OUD scale was administered twice to a subsample of the general population who agreed to be contacted by phone. At least a one-month interval (with a maximum of three months) separated each call.

#### Population with OUD (sample 2)

The second step involved assessing the criterion validity of the ORT-OUD. The Arabic versions of the ORT-OUD and the ASSIST-opioid subscale were used to evaluate the risk of developing OUD in a clinical sample of patients previously diagnosed with OUD and treated for this disorder. The recruitment took place at the Skoun addiction center, which offers a free-of-charge program in Beirut, Lebanon. All patients (46 patients in total) who were present at Skoun during the inclusion period (between May 2022 and July 2022) were invited to join the study and fill out the questionnaire. Patients had to meet inclusion criteria, i.e., having a diagnosis of OUD, being over 18, being Lebanese, providing consent to complete the questionnaire, and being fluent in Arabic. The diagnosis of OUD had been previously established by a psychiatrist through a clinical evaluation following the criteria outlined in the Diagnostic and Statistical Manual of Mental Disorders, Fifth Edition (DSM-5).

Patients were asked to fill out a paper version of the questionnaire to enhance the completion of the survey and were supported by a research assistant who ensured that all questions were addressed; of note, the research assistant did not interfere during the process, except for providing guidance to participants in completing the questionnaire.

### Sample size calculation

Comrey and Lee suggested a minimum of ten observations per variable to perform an exploratory factor analysis [30] when assessing construct validity. Since the revised ORT is a 9-item questionnaire, a minimum of 90 patients was required for this study.

For the epidemiological study, the minimum sample size was calculated using the Epi-info software. The expected frequency was kept at 50% to yield the largest sample size. Accordingly, a sample of 384 participants was required to produce a 95% confidence interval, with a 5% alpha error and a power of 80%.

### Questionnaire

The online questionnaire was available in English and Arabic, the native language in Lebanon (Appendix 1), and consisted of four parts. The first assessed the sociodemographic characteristics of the participants, including age, gender, weight, height, marital status, nationality, highest educational level, employment status and occupation, religion, current household monthly income, socioeconomic status, and medical history of chronic and mental illness. The socioeconomic status was assessed using the crowding index (calculated by dividing the number of individuals living in the house by the number of rooms), which was then categorized into quartiles. Other questions were related to medical coverage, smoking and alcohol consumption, and self-perception of the financial situation.

The second part of the questionnaire consisted of two validated scales for the evaluation of substance use disorders, i.e., the Alcohol, Smoking, and Substance Involvement Screening Test (ASSIST) [24] and the Opioid Risk Tool Revised (ORT-OUD) [27], which was translated into Arabic and validated.

The third part consisted of several validated scales to assess sleep disorders: the Pittsburgh Sleep Quality Index (PSQI) [31], the Insomnia Severity Index (ISI) [32], and the Epworth Sleepiness Scale (ESS) [33]. The chronotype of the participants was also evaluated using the Composite Scale (CS) [34]. The final part included the Hospital Anxiety and Depression Scale (HADS) used to assess depression (HADS-D) and anxiety (HADS-A) [35]. Permission from copyright holders was obtained to use the validated scales.

#### The alcohol, smoking, and substance involvement screening test – opioid subscale (opioid ASSIST; arabic version)

ASSIST is an 8-item tool developed by the World Health Organization (WHO) to screen for substance use-related health risks and substance use disorders in primary care and other settings [24]. It assesses the risk related to different substances, such as tobacco products, alcohol, cannabis, cocaine, amphetamine-type stimulants (ATS), sedatives and sleeping pills (benzodiazepines), hallucinogens, inhalants, opioids, and “other” drugs and is available in Arabic [36]. Only three subscales were used in this study: opioids, sedatives or sleeping pills, and alcoholic beverages. This selection was based on existing literature that has shown a correlation between OUD mainly and sedatives and alcohol use disorders [37, 38]. Each item was weighted differently, and higher total subscores predicted higher risks of developing related substance use disorder. It also indicated if the participant was at low (0–3 for opioids and sedatives subscores; 0–10 for alcohol subscore), moderate (4–24 for opioids and sedatives subscores; 11–26 for alcohol subscore), or high risk (27 + for all subscores) of experiencing severe problems resulting from the current pattern of use.

#### The revised opioid risk tool (ORT-OUD)

The ORT consists of ten weighted items and is used to rapidly screen for the risk of developing OUD. According to the total score, participants are classified into potential high, moderate, or low-risk level. A simplified version, the ORT-OUD, was used in this study. It was created by unweighting all items and reducing their number to nine by removing one item related to preadolescent sexual abuse. Answers are scored on a dichotomous Yes/No scale. A cut-off point of 2.5 was adopted; scores of 0, 1, or 2 indicate a non-OUD status, while scores ≥ 3 suggest potential risk for OUD. The selected cut-off score of 2.5 was based on the excellent sensitivity and specificity demonstrated in the initial development and validation study of the ORT-OUD [27].

#### The pittsburgh sleep quality index (PSQI)

The PSQI is a self-report questionnaire designed to assess sleep quality and sleep disorders over a one-month period. It consists of 19 items that generate seven component scores: subjective sleep quality, sleep latency, sleep duration, habitual sleep efficiency, sleep disturbances, use of sleeping pills, and daytime dysfunction. The total score is calculated by summing the scores of these seven components. The higher the score, the worse the sleep quality [31].

#### The insomnia severity index (ISI)

The ISI is a self-report instrument used to measure patient perceived insomnia. It targets the subjective symptoms and consequences of insomnia and the level of worry or distress caused by these difficulties. The total score enables to determine the presence and severity of insomnia. Values between 0 and 7 indicate the absence of insomnia, 8–14 sub-threshold insomnia, 15–21 moderate insomnia, and 22–28 severe insomnia [32].

#### The epworth sleepiness scale (ESS)

The ESS is a subjective measure of sleepiness widely used in sleep medicines. It consists of a list of eight situations where individuals rate their tendency to doze on a scale from 0 (no chance of dozing) to 3 (high chance of dozing). According to scores, sleepiness is categorized as normal (0–10), mild (11–14), moderate (15–17), and severe (18–24) [33].

#### The composite scale (CS)

The CS is a 13-question tool used to evaluate the chronotype, which refers to the general preferences regarding the timing of waking up, falling asleep, and peak performance [34]. The scores range from 1 to 4 or 5, depending on the question. Scores can indicate an evening circadian typology (22 or less), morning circadian typology (higher than 44), or intermediate circadian typology (between 22 and 44) [39].

#### Hospital anxiety and depression scale (HADS)

The HADS is a 14-item self-report scale consisting of two subscales of seven items each, designed to assess anxiety (HADS-A) and depression (HADS-D). The total score for each subscale is the sum of the seven items (ranging from 0 to 21). A score between 0 and 7 indicates no anxiety or depression, while scores of 8 to 10 suggest mild anxiety or depression, 11 to 14 moderate anxiety or depression, and 15 to 21 severe anxiety or depression [35].

### Validation and piloting of the ORT-OUD

The translation procedure started after getting the approval from Professor Martin Cheatle, the author of the scale. The ORT-OUD underwent an initial translation from English into Arabic, followed by a validation process through back-translation (Additional File). Independent professional translators conducted both the translation and back-translation. The research team and translators compared the original English version with the back-translated version to ensure that the questions had the same meaning, making the necessary corrections as needed. Cultural adaptation of the items was not performed during this process. The scale was then piloted following the finalization of the translation. It was administered to 56 bilingual participants in both languages. Its reliability was found to be excellent, with a single measures intraclass correlation coefficient of 0.95 and an average measures intraclass coefficient of 0.974 [40]. The final version of the questionnaire was deemed easy to understand and complete. A pilot test was conducted among ten individuals who were not part of the study to ensure the clarity of the questions. Based on their feedback, one question in the sociodemographic section was reformulated for better comprehension. The responses from the pilot study were not included in the final database.

### Statistical analysis

For the general population (sample 1), data from Google Forms were generated and collected on Excel sheets and then transferred to IBM SPSS^®^ software version 25.0 for analyses. Descriptive statistics were calculated for all variables in the study. The Kolmogorov-Smirnov test was used to verify the normality of the continuous variables. Means and standard deviations were shown for normally distributed variables, and medians and interquartile ranges for non-normally distributed variables. Frequencies and percentages were displayed for dichotomous and multinomial variables.

A factor analysis was conducted using the principal component analysis (PCA) technique to evaluate the construct validity of the ORT-OUD scale. This analysis was performed on the nine items of the ORT-OUD scale, and a Varimax rotation was applied since the extracted factors were not found to be significantly correlated. The Kaiser-Meyer-Olkin (KMO) measurement and the Bartlett sphericity test were performed to ensure the sampling adequacy. The number of factors corresponding to Eigenvalues greater than one were retained.

Cronbach’s alpha coefficient was calculated for the reliability analysis of the total score: α ≥ 0.7 and ≥ 0.8 reflected acceptable and excellent internal consistency values, respectively [41]. The test-retest reliability was evaluated by the intraclass correlation coefficient (ICC, mean measurement) for the scores of participants with repeated measures. ICC values less than 0.5 suggest poor reliability, values between 0.5 and 0.75 indicate moderate reliability, values between 0.75 and 0.9 indicate good reliability, and values greater than 0.9 suggest excellent reliability [42].

Bivariate and multivariable analyses were performed for sample 1 (general population), taking the opioid use risk scores as dependent variables. The association between continuous variables was verified using the Pearson’s correlation test in bivariate analysis. The Student’s t-test was used to compare means for two groups, while ANOVA was used for three groups and more when the continuous variable followed a normal distribution. The Mann-Whitney test was used for comparing two groups, and the Kruskal-Wallis test was used for comparing three or more groups when the continuous variable did not follow a normal distribution. The Chi-Squared test was utilized to compare percentages when all expected values were greater than 5, and the Fisher’s exact test was used when at least one expected value was lower than 5. Finally, multivariable analyses were conducted to account for potential confounding factors. A value of p ≤ 0.05 was considered statistically significant.

## Results

Sample 1 consisted of 581 participants from different regions of Lebanon, with 68.8% female (n = 400) and 31.2% male (n = 181). The mean age was 25.29 ± 8.1 years (mean ± standard deviation). Of the total participants, 67.8% (n = 394) were single, 29.6% (n = 172) were married, and 91.6% (n = 532) had a university level of education. Sample 2 comprised 46 patients with a previous diagnosis of OUD. Table 1 summarizes the sociodemographic characteristics of the included population.

**Table 1:** Sociodemographic and other characteristics of the patients

		Frequency (%)	
		General population (Sample 1) (N = 581)	Opioid use disorder patients (Sample 2) (N = 46)
Gender	Male	181 (31.2%)	44 (95.7%)
	Female	400 (68.8%)	2 (4.3%)
Marital Status	Married	172 (29.6%)	27 (26.1%)
	Single	394 (67.8%)	15 (63%)
	Divorced	5 (0.9%)	5 (10.9%)
Family income (in LBP)	< 3 millions	192 (33.0%)	30 (65.2%)
	> 3 millions	287 (49.4%)	1 (2.2%)
	Prefer not to answer	102 (17.6%)	15 (32.6%)
Highest level of education	No education	0 (0%)	5 (10.9%)
	Complementary	14 (2.4%)	9 (19.6%)
	Primary	1 (0.2%)	6 (13%)
	Secondary	34 (5.9%)	13 (28.3%)
	University	532 (91.6%)	13 (28.3%)
Occupation	Do not work	42 (7.2%)	3 (6.5%)
	Currently unemployed	214 (36.8%)	15 (32.6%)
	Healthcare professional	141 (24.3%)	0 (0%)
	Employed	184 (31.7%)	28 (60.9%)
Christian religion	No	195 (33.6%)	41 (89.1%)
	Yes	386 (66.4%)	5 (10.9%)
Alcohol consumption	No	281 (48.4%)	37 (80.4%)
	Yes	300 (51.6%)	9 (19.6%)
Cigarette smoking	No	457 (78.7%)	0 (0%)
	Yes	124 (21.3%)	46 (100%)
Family history of chronic disease	No	463 (79.7%)	35 (76.1%)
	Yes	118 (20.3%)	11 (23.9%)
Family history of neuropsychiatric disease	No	536 (92.3%)	26 (56.5%)
	Yes	45 (7.7%)	20 (43.5%)
**Continuous variables**		**Mean ± SD**	
Age (in years)		25.29 ± 8.08	39.89 ± 10.9
Weight (in Kg)		75.88 ± 18.42	72.67 ± 12.34
Height (in cm)		170.82 ± 10.19	175.7 ± 8.1
Number of cigarettes per day		14.47 ± 10.68	24.61 ± 11.3
***Sleep disorders***			
PSQI		6.41 ± 3.67	
ISI		7.53 ± 4.49	
ESS		46.41 ± 9.18	
***Chronotype***			
CS		8.65 ± 4.27	
***Mood disorders***			
HADS-A		10.41 ± 4.18	
HADS-D		7.65 ± 3.10	

***Abbreviations***: *CS: Composite scale; ESS: The Epworth Sleepiness Scale; HADS-A: Hospital Anxiety and Depression Scale (Anxiety subscale); HADS-D: Hospital Anxiety and Depression Scale (Depression subscale); ISI: Insomnia severity index; LBP: Lebanese Pounds.*

### Validation of the ORT-OUD scale

The PCA of the ORT-OUD scale was run over sample 1 (n = 581). None of the scale items was removed; the items converged to a solution of four factors with an Eigenvalue over 1, explaining a total of 68.2% of the variance. The four factors were: History of substance abuse (3 items), history of alcohol abuse (2 items), history of illegal drug abuse (2 items), and psychological factors (2 items). Table 2 displays items’ loading. A Kaiser-Meyer-Olkin measure of sampling adequacy of 0.632 was found, with a significant Bartlett’s test of sphericity (*p* < 0.001). Communalities for the ORT-OUD items were obtained and are detailed as supplementary data.

**Table 2:** Results of the Varimax rotated matrix of the ORT-OUD items

	Components			
	Factor 1: History of substance abuse	Factor 2: History of alcohol abuse	Factor 3: History of illegal drug abuse	Factor 4: Psychological factors
Personal history of substance abuse (prescription drugs)	0.838			
Family history of substance abuse (prescription drugs)	0.834			
Personal history of substance abuse (age between 16–45 years)	0.507			
Family history of substance abuse (alcohol)		0.854		
Personal history of substance abuse (alcohol)		0.828		
Family history of substance abuse (illegal drugs)			0.819	
Personal history of substance abuse (illegal drugs)			0.813	
Psychological disease (ADD, OCD, bipolar disorder, schizophrenia)				0.865
Psychological disease (depression)				0.668

***Abbreviations***: *ADD: Attention Deficit Disorder; OCD: Obsessive Compulsive Disorder; ORT-OUD: Opioid Risk Tool revised.*

### Cronbach’s alpha values and ICC between the test and retest

The Cronbach’s alpha value was moderate (0.648). The Interclass Correlation Coefficients (ICC) for the total ORT-OUD score were valued at 0.644 for single measures (*p* < 0.001) and 0.784 for average measures (*p* < 0.001).

### Description of the ORT-OUD scale scores

Table 3 describes the ORT-OUD scale score and the ASSIST subscales (opioids, sedatives, and alcohol) in the general population (n = 581) and among patients with OUD (n = 46).

**Table 3:** Mean scores of the ORT-OUD total score and ASSIST subscale scores

Total Score	Mean ± SD	
	General population (n = 581)	OUD patients (n = 46)
**ORT-OUD**	1.94 ± 0.406	4.26 ± 1.389
**ASSIST-opioids**	0.18 ± 0.176	9.24 ± 8.561
**ASSIST-sedatives**	0.71 ± 0.513	7.93 ± 9.453
**ASSIST-alcohol**	8.29 ± 1.516	2.39 ± 5.026

***Abbreviations***: *ASSIST-alcohol*: *Alcoholic beverages subscale of the Alcohol, Smoking, and Substance Involvement Screening Test; ASSIST-opioid*: *Opioid subscale of the Alcohol, Smoking, and Substance Involvement Screening Test; ASSIST-sedatives: Sedatives and sleeping pills subscale of the Alcohol, Smoking, and Substance Involvement Screening Test; ORT-OUD: Revised Opioid Risk Tool; SD*: *Standard deviation.*

### Convergence between ORT-OUD and ASSIST subscale scores

The correlation coefficients between the ORT-OUD total score and the ASSIST subscale scores (opioids, sedatives, and alcohol) in the general population (n = 581) and among OUD patients with (n = 46) showed that ORT-OUD correlated positively (p < 0.001) with all the ASSIST subscales in the general population. However, in OUD patients, a significant correlation was noted only with the opioid (r = 0.251, p = 0.093) and the sedatives subscales (r = 0.598, p < 0.001), but not the alcohol subscale. Results are summarized in supplementary data.

### Bivariate analysis (sample 1: general population)

The bivariate analysis, taking the ORT-OUD as the dependent variable, showed that higher ORT-OUD scores were associated with a higher number of glasses of alcohol consumed per occasion, higher scores of all three ASSIST subscales (opioids, sedatives, and alcohol) and more sleep disorders (as evaluated by PSQI, ESS, and ISI) and mental disorders (higher HADS-A and HADS-D scores). When taking the ASSIST-opioids subscale as the dependent variable, a higher opioid risk was noted with higher scores of sedatives/hypnotics and the ASSIST-alcohol subscale, but also with more severe insomnia (higher ISI scores). Details are presented in Tables 4, 5 and 6.

**Table 4:** Bivariate analyses of the sociodemographic parameters considering ORT-OUD and ASSIST-Opioid scores as the dependent variables in the general population

	ORT-OUD			ASSIST-Opioid		
Sociodemographic parameters	Mean ± SD	Median [M-M]	*p*-value**	Mean ± SD	Median [M-M]	*p*-value**
**Gender**						
Male	1.25 ±0.648	0.50 [0–5]	0.109	0.38 ±0.375	0.00 [0–3]	0.179
Female	2.56 ±0.444	3.00 [0–4]		0.00±0.000	0.00 [0–3]	
**Monthly family income (in LBP)**						
Less than 3 millions	3.00±0.730	3.50 [0–5]	0.168	0.00±0.000	0.00 [0–0]	0.254
More than 3 millions	1.22±0.465	1.00 [0–3]		0.33±0.333	0.00 [0–0]	
Prefers not to say	2.00±1.000	2.00 [1–3]		0.00±0.000	0.00 [0–0]	
**Occupation**						
Doesn’t work	2.25 ±0.750	2.00 [1–4]	**< 0.001**	0.00±0.000	0.00 [0–0]	0.531
Healthcare worker	0.75±0.750*	0.00 [0–3]		0.00±0.000	0.00 [0–0]	
Unemployed	2.17±0.601	2.50 [0–4]		0.50±0.500	0.00 [0–3]	
Employee	2.67±1.453	3.00 [0–5]		0.00±0.000	0.00 [0–0]	
**Governorate**						
Beirut	1.67±1.202	1.00 [0–4]	0.794	0.00±0.000	0.00 [0–0]	**0.033**
Mount Lebanon	1.80±0.593	1.50 [0–5]		0.30±0.300*	0.00 [0–3]	
Others	2.50±0.500	3.00 [1–3]		0.00±0.000	0.00 [0–0]	
**University education**						
Yes	1.67±0.398	1.00 [0–4]	0.545	0.00±0.200	0.00 [0–3]	**0.038**
No	4.00±1.000	4.00 [3–5]		0.00±0.000	0.00 [0–0]	
**Religion**						
Christian	2.67±0.577	3.00 [0–5]	**0.006**	0.00±0,000	0.00 [0–0]	**0.002**
Others	1.13±0.441	1.00 [0–3]		0.38±0,375	0.00 [0–3]	
**Socioeconomic quartiles**						
≤ 1,00	2.13±0.666	2.00 [0–5]	0.242	0.38±0.375	0.00 [0–3]	0.773
1.01–1.25	1.67±0.882	2.00 [0–3]		0.00±0.000	0.00 [0–0]	
1.26–1.67	2.33±1.202	3.00 [0–4]		0.00±0.000	0.00 [0–0]	
1.68 +	1.33±0.882	1.00 [0–3]		0.00±0.000	0.0 0–0]	

*Indicates the modality that significantly differs from the others; **Numbers in bold represent statistically significant values.

***Abbreviations***: *ASSIST-Opioid: Opioid subscale of the Alcohol, Smoking, and Substance Involvement Screening Test; M-M: Minimum-Maximum; ORT-OUD: Revised Opioid Risk Tool; SD: Standard deviation.*

**Table 5:** Bivariate analyses of continuous variables considering ORT-OUD and ASSIST-Opioid scores as the dependent variables in the general population

	ORT-OUD		ASSIST-Opioid	
Continuous variable	Correlation coefficient -r-	*p-*value*	Correlation coefficient -r-	*p-*value*
ֻAge (year)	-0.048	0.244	0.050	0.232
Weight (Kg)	0.076	0.067	0.061	0.140
Height (cm)	0.004	0.924	0.001	0.987
Number of cigarettes per day	0.132	0.159	0.097	0.299
Number of waterpipes per week	0.033	0.737	0.108	0.272
Number of glasses of alcohol per occasion	0.136	**0.018**	-0.006	0.919

*Numbers in bold represent statistically significant values.

***Abbreviations***: *ASSIST-Opioid: Opioid subscale of the Alcohol, Smoking, and Substance Involvement Screening Test; ORT-OUD: Revised Opioid Risk Tool; SD: Standard deviation.*

**Table 6:** Bivariate analyses with the scale scores considering ORT-OUD and ASSIST-Opioid scores as the dependent variables in the general population

	ORT-OUD		ASSIST-Opioid	
	Correlation coefficient -r-	*p*-value*	Correlation coefficient -r-	*p*-value*
ASSIST-opioids	0.174	**< 0.001**		
ASSIST-sedatives	0.249	**< 0.001**	0.625	**< 0.001**
ASSIST-alcohol	0.161	**< 0.001**	0.213	**< 0.001**
ORT-OUD			0.174	**< 0.001**
PSQI	0.208	**< 0.001**	0.039	0.344
ISI	0.271	**< 0.001**	0.096	**0.021**
ESS	0.121	**0.004**	0.017	0.674
CS	-0.022	0.594	0.041	0.325
HADS-A	0.124	**0.003**	0.001	0.976
HADS-D	0.223	**< 0.001**	0.054	0.191

*Numbers in bold represent statistically significant values.

***Abbreviations***: *ASSIST-alcohol*: *Alcoholic beverages subscale of the Alcohol, Smoking, and Substance Involvement Screening Test; ASSIST-opioid*: *Opioid subscale of the Alcohol, Smoking, and Substance Involvement Screening Test; ASSIST-sedatives: Sedatives and sleeping pills subscale of the Alcohol, Smoking, and Substance Involvement Screening Test; CS: Composite scale; ESS: The Epworth Sleepiness Scale; HADS-A: Hospital Anxiety and Depression Scale (Anxiety subscale); HADS-D: Hospital Anxiety and Depression Scale (Depression subscale); ISI: Insomnia severity index; LBP: Lebanese pounds; OUD: Opioid Use Disorder*, *ORT-OUD: Revised Opioid Risk Tool; PSQI: Pittsburgh Sleep QualityIndex; SD*: *Standard deviation.*

### Multivariate analyses (sample 1: general population)

Multivariate analyses, taking the ORT-OUD score as the dependent variable, showed that the ORT-OUD score was positively and significantly correlated with a family history of alcohol abuse (B = 0.895), illegal drug use (B = 1.02), and prescription drugs (B = 1.10) and a personal history of alcohol abuse (B = 1.10), illegal drug use (B = 1.05), and prescription drugs (B = 1.08). A positive correlation was also noted with age (if the participant was between 16 and 45 years old; B = 1.04), with higher ISI (B = 0.009) and HADS-A scores (B = 0.010). This score was significantly higher in people with psychiatric illnesses, such as obsessive-compulsive disorder (OCD), post-traumatic stress disorder (PTSD), schizophrenia, and bipolar disorder (B = 1.28), while it was negatively correlated with the number of waterpipes consumed per week (B=-0.015) (Table 7; Model 1).

**Table 7:** Multivariate analysis considering the opioid use disorder score as the dependent variable

**Model 1: Multivariate analysis considering the ORT-OUD score as the dependent variable**					
**Model**	**Unstandardized coefficients**		***p-*** value*	**95% CI**	
**Variable**	**B**	**Standard error**		**Lower Bound**	**Upper Bound**
Religion: Others	0.074	0.0303	**0.015**	0.014	0.133
Family history of alcohol abuse	0.895	0.0460	**< 0.001**	0.805	0.985
Family history of illegal drug use	1.02	0.1274	**< 0.001**	0.778	1.277
Family history of prescription drug abuse	1.10	0.0487	**< 0.001**	1.007	1.198
Personal history of alcohol abuse	1.10	0.0520	**< 0.001**	1.006	1.210
Personal history of illegal drug use	1.05	0.1219	**< 0.001**	0.819	1.297
Personal history of prescription drug use	1.08	0.0628	**< 0.001**	0.958	1.205
Age between 16–45 years old	1.04	0.0342	**< 0.001**	0.981	1.116
Psychiatric diseases	1.28	0.0639	**< 0.001**	1.163	1.414
Consumption of waterpipe	-0.015	0.0068	**0.023**	-0.029	-0.002
HADS-A score	0.010	0.0039	**0.012**	0.002	0.017
ISI score	0.009	0.0037	**0.014**	0.002	0.016
Variables entered in the model: gender, occupation, family income, governate, education, marital status, religion, socioeconomical quartiles, family history of substance abuse, family history of substance abuse (illegal drugs), family history of substance abuse (prescription drugs), personal history of substance abuse (alcohol), personal history of substance abuse (illegal drugs), personal history of substance abuse (prescription drugs), age between 16–45 years, Psychological disease (Attention deficit disorder (ADD), obsessive-compulsive disorder (OCD), bipolar disorder, schizophrenia), age, weight, height, number of cigarettes per day, number of waterpipes per week, number of alcohol glasses per occasion, HADS-A score, HADS-D score, PSQI score, ISI score, CS score, ESS score.					
**Model 2: Multivariate analysis considering the ASSIST-opioids score as the dependent variable**					
**Model**	**Unstandardized coefficient**		***p-*** value*	**95% CI**	
**Variable**	**B**	**Standard error**		**Lower Bound**	**Upper Bound**
Family history of illegal drug use	2.366	0.9548	**0.013**	0.494	4.237
Personal history of illegal drug use	3.53	0.9139	**< 0.001**	1.745	5.327
Personal history of prescription drug use	1.52	0.4708	**0.001**	0.597	2.443
Number of glasses of alcohol per occasion	-0.57	0.0660	**0.017**	0.028	0.286
ISI score	0.094	0.0278	**0.001**	0.040	0.149
Variables entered in the model: gender, occupation, family income, governate, education, marital status, religion, socioeconomical quartiles, family history of substance abuse, family history of substance abuse (illegal drugs), family history of substance abuse (prescription drugs), personal history of substance abuse (alcohol), personal history of substance abuse (illegal drugs), personal history of substance abuse (prescription drugs), age between 16–45 years, Psychological disease (Attention deficit disorder (ADD), obsessive-compulsive disorder (OCD), bipolar disorder, schizophrenia), age, weight, height, number of cigarettes per day, number of waterpipes per week, number of alcohol glasses per occasion, HADS-A score, HADS-D score, PSQI score, ISI score, CS score, ESS score.					

*Numbers in bold represent statistically significant values.

***Abbreviations***: *B: Unstandardized regression coefficient; CI: confidence interval; ORT-OUD: Revised Opioid Risk Tool; ASSIST-Opioid: Opioid subscale of the Alcohol, Smoking, and Substance Involvement Screening Test; ISI: Insomnia severity Index; HADS-A: Hospital Anxiety and Depression Scale (Anxiety subscale).*

A second multivariate analysis taking the ASSIST-opioids score as the dependent variable showed that the ASSIST-opioids score was significantly and positively correlated with a family history of illegal drug use (B = 2.36), a personal history of prescription (B = 1.52) and illegal drug use (B = 3.53), and ISI scores (B = 0.094), while it was negatively correlated with the number of alcohol glasses per week (B=-0.157) (Table 7; Model 2).

## Discussion

In this study, the ORT-OUD was translated into Arabic and validated in the general population (sample 1), and its criterion validity was confirmed in a clinical sample of participants with OUD (sample 2). Construct validity analysis resulted in the distribution of items on four factors, i.e., history of substance abuse, history of alcohol abuse, history of illegal drug use, and psychological factors. These factors demonstrated rational explanations, appropriate sampling adequacy, anti-image correlations, and communalities.

It is worth noting that the initial validation of the ORT-OUD scale in the original paper determined the discriminant predictive validity and the receiver operating characteristic (ROC) curve in two samples of chronic nonmalignant pain patients taking long-term opioid therapy; the first sample of patients developed OUD after starting opioid therapy, while the other one displayed no evidence of OUD. However, it did not include a factor analysis, which makes direct comparison with the present study’s results challenging. Furthermore, to the best of our knowledge, no previous research has conducted the translation and validation of the ORT-OUD scale.

Reliability analysis in this study revealed a Cronbach’s alpha value of 0.648, suggesting acceptable internal consistency reliability [43]. Nevertheless, the average ICC between the test and retest indicated good reliability [42].

The ORT-OUD score converged well with the ASSIST subscales of sedatives/hypnotics and alcoholic beverages, confirming the association between opioid use risk and other substance use disorders.

Firstly, the ORT-OUD score correlated positively with the sedatives and hypnotics subscales of the ASSIST tool, consistent with previous findings showing that 70% of OUD patients reported lifetime use of nonmedical sedatives and tranquilizers and 11.3% had a sedative/tranquilizer use disorder [38]. OUD patients who use nonmedical sedatives and tranquilizers often exhibit higher rates of polysubstance use and other substance use disorders [44, 45]. This correlation highlights the potential risk of drug-drug interactions and fatal opioid overdoses associated with the simultaneous consumption of opioids and sedatives such as benzodiazepines [46]. Moreover, several studies among OUD patients have found correlations between the use of nonmedical sedatives and tranquilizers and sociodemographic characteristics, such as female gender, younger age, and indicators of opioid use severity [47–49]. These characteristics have been associated with a higher likelihood of using nonmedical sedatives and tranquilizers, making them essential to understanding the patterns of substance use and polysubstance use among individuals with OUD.

Secondly, the ORT-OUD score also correlated with the ASSIST-alcohol subscale in the general population, in line with findings from studies conducted in the United States suggesting a higher risk of concurrent Alcohol Use Disorder (AUD) in adults with nonmedical prescription opioid use and OUD [50]. Another study among OUD patients revealed that 23.4% of the sample had a co-diagnosis of AUD [51].

Lastly, ORT-OUD demonstrated associations with the opioid subscale of the ASSIST tool in the general population, highlighting its ability to identify similar patterns of substance use as the comprehensive ASSIST scale. This finding indicates good convergent validity of the Arabic version of the ORT-OUD, affirming its usefulness in assessing opioid use risk and its relationship with other substance use disorders. However, the correlation of ORT-OUD with the opioid subscale of the ASSIST tool was inconclusive among OUD patients, likely due to the small sample size.

By employing both the Arabic ORT-OUD scale and the ASSIST-opioid subscale, the prevalence of OUD was estimated at 14.5% and 6.54%, respectively. The difference in prevalence rates between the two scales can be attributed to their different approaches to assessing OUD risk. Indeed, ORT-OUD evaluates the risk of developing OUD by considering various well-known risk factors such as age, family, personal history of substance abuse, and mental health conditions, while the ASSIST-opioid subscale focuses more on current substance use habits, thus leading to lower estimated prevalence [21, 24].

Other studies using the ORT tool have reported a risk prevalence of developing OUD ranging from 9 to 11.6% [52, 53]. Interestingly, one of these studies evaluated the association between hurricane exposure and the risk of opioid-abusive behavior. The findings indicated that exposure to a natural disaster, particularly personal exposure, was associated with an increased risk of opioid-abusive behavior, with approximately 9% of participants classified as having a high risk of developing an OUD [52]. Our study was conducted in the context of multiple crises, including the COVID-19 pandemic, an economic collapse described as one of the worst crises of the past century [15, 54], and the devastating Beirut port explosion in August 2020 considered one of the largest non-nuclear blasts ever recorded in history, with more than 200 deaths, 7000 injured, and 300,000 displaced Lebanese citizens [14, 15, 55]. Nevertheless, as data on OUD before these crises are lacking, no conclusions can be drawn as to whether these crises might have potentially affected the high prevalence values reported in this paper; more robust, larger-size epidemiological studies would provide a better understanding of prevalence trends over time.

Another valuable factor is the lack of comprehensive evaluation and follow-up by healthcare practitioners when prescribing opioids. It has been reported that a significant number of healthcare practitioners do not thoroughly assess patients for potential risk factors before prescribing opioids in Lebanon. Additionally, inadequate follow-up and insufficient communication about possible adverse effects are common among healthcare practitioners in Lebanon. This lack of proper evaluation and follow-up can contribute to the emergence of an opioid epidemic by increasing the likelihood of people developing OUD [17].

This study also aimed to explore the association between sociodemographic and clinical data and the risk of OUD in the Lebanese population. The results showed that having a family and a personal history of illegal/prescription drug use increases the predisposition to develop OUD. It is well-established that a family history of substance use disorder is a risk factor for OUD in patients with chronic nonmalignant pain [56–60]. Furthermore, research has demonstrated that teenagers with a family history of alcohol or drug abuse and a lack of pro-social skills are more prone to transition quickly from occasional use to severe patterns of abuse or dependence [61]. Thus, understanding these factors helps elucidate the etiopathology and trajectory of addictive behaviors. Finally, social risk factors, such as connection with deviant peers, popularity, bullying, and gang affiliation, can help in shaping positive beliefs and attitudes toward drug use. Therefore, friends and family provide immediate access to substances and also serve as role models for behavior and drug use [62, 63].

When exploring sleep patterns, a positive and significant correlation was observed between the risk of developing OUD and sleep disorders, as evaluated by the ISI. The association between illegal psychoactive substance use and sleep problems appears to be bidirectional [64]. Sleep problems have been found to increase the risk of developing substance use disorders [65–67], which, in turn, might lead to sleep problems [67–70]. Evidence suggests that chronic use of some illicit substances results in chronic sleep alterations, distinct from the acute effects of these substances [71]. A recent study exploring the bidirectional relationship between lack of sleep and the need to use opioids found that opioid craving/use and sleep deficiency share common circuits linked via the activation of stress-regulatory systems, such as the sympathetic nervous system, the hypothalamic-pituitary-adrenal axis, and inflammatory processes [72]. Another study has even shown that suvorexant, an orexin-blocking sleep medication approved for the treatment of insomnia, can also decrease opioid-induced cravings [73].

Our study is the first to assess the relationship between chronotype and the risk of OUD in the Lebanese population. While it yielded inconclusive results with the Composite Scale used to evaluate chronotype, other studies found a connection between circadian preferences, such as eveningness, and substance use disorder in young adults and adolescents [74, 75].

Regarding mood and other psychiatric disorders, our results revealed that individuals with high anxiety scores (as evaluated by the HADS-A) and those with psychiatric illnesses were more likely to develop OUD. A strong association exists between opioid- and anxiety-related symptoms and disorders [76], which are more common and more strongly associated with the use of prescribed opioids than other substances [76–78]. Furthermore, individuals with a genetic predisposition for OUD are at increased risk of developing anxiety, stress-related disorders, and major depressive disorder [79]. Common mental health disorders and problematic drug use have been found to be associated with the initiation and use of prescribed opioids in the general population [80]. Therefore, it is essential to accurately evaluate and identify psychiatric disorders before starting an opioid treatment for pain management [81].

Finally, our study revealed that higher waterpipe use was linked to a lower risk of developing OUD, probably because the high nicotine content of waterpipe smoke helps reduce anxiety [82], thereby decreasing the need to seek drugs, including opioids. Additionally, anxious individuals may have difficulty self-regulating during stressful situations and may turn to external methods, such as tobacco use, to cope with stress [83].

### Limitations and strengths

This study has several limitations. Other scales could have been used to compare the results obtained with the ORT-OUD, such as the Screener and Opioid Assessment for Patients with Pain-Revised (SOAPP^®^-R) [84] or the Diagnosis, Intractability, Risk, Efficacy (DIRE) scale [26], as the ASSIST-opioids subscale might not be the best tool to evaluate the risk of developing OUD. However, it was selected because it is the only scale with complete validity and reliability data in Arabic [85]. Other limitations are related to the demographic characteristics of the general population (sample 1) since more than 60% of participants were female, young, with a university level of education and good computer literacy. Thus, our results may not be generalized to the entire population. Finally, due to the presence of multiple crises, including the COVID-19 pandemic and the massive Beirut port explosion, the prevalence values reported should be interpreted with caution, as these external factors may have influenced the results. Additionally, the lack of data related to OUD before these crises limits our ability to draw definitive conclusions about the specific influence of these factors.

Despite all these limitations, this study is the first to validate an OUD questionnaire in the Lebanese population. This validated tool can now be used in any Arabic-speaking country to assess the risk of OUD before initiating opioid therapy. Moreover, our study is the first nationwide and regional investigation of OUD and potential risk factors, such as sleep disorders, chronotype, and mood disorders.

## Conclusions

This study validated the Arabic version of the ORT-OUD scale, confirming its validity and reliability in the Lebanese population. By taking into account modifiable risk factors such as insomnia and anxiety, this scale can help identify people at risk of developing OUD, allowing for targeted interventions to reduce the risk of OUD and improve patient outcomes. The validation of the Arabic version of the ORT-OUD scale in the Lebanese population is a milestone in improving the detection and management of OUD in this population.

### Electronic supplementary material

Below is the link to the electronic supplementary material.

Additional file 1 - Supplementary Table 1. Communalities for the ORT-OUD items. Supplementary Table 2. Correlation coefficients between the ORT-OUD total score and the ASSIST subscales scores.

## Data Availability

All data and materials are available upon request to corresponding author.

## Abbreviations

ASSIST-opioid: Opioid subscale of the Alcohol, Smoking, and Substance Involvement Screening Test
ASSIST-sedatives: Sedatives and sleeping pills subscale of the Alcohol, Smoking, and Substance Involvement Screening Test
AUD: Alcohol Use Disorder
CS: Composite scale
DSM-5: Diagnostic and Statistical Manual of Mental Disorders, Fifth Edition
ESS: The Epworth Sleepiness Scale
GSHS: Global School Health Survey
HADS-A: Hospital Anxiety and Depression Scale (Anxiety subscale)
HADS-D: Hospital Anxiety and Depression Scale (Depression subscale)
ISI: Insomnia severity index
KMO: Kaiser-Meyer-Olkin
LBP: Lebanese pounds
OCD: Obsessive-Compulsive Disorder
OUD: Opioid Use Disorder
ORT-OUD: Opioid Risk Tool revised
PCA: Principal Component Analysis
PTSD: Post-Traumatic Stress Disorder
PSQI: Pittsburgh Sleep Quality Index
