# Supplementary material for: Opioid use disorder in two samples of the Lebanese population: scale validation and correlation with sleep and mood disorders
Source: BMC Psychiatry. 2023 Nov 1;23:797. doi: 10.1186/s12888-023-05304-8 (PMC10619223; doi:10.1186/s12888-023-05304-8)
Supplement: Supplementary file 1 — Additional file 1 - Supplementary Table 1. Communalities for the ORT-OUD items. Supplementary Table 2. Correlation coefficients between the ORT-OUD total score and the ASSIST subscales scores. [file 12888_2023_5304_MOESM1_ESM.docx]

# Additional File 1

# Supplementary Tables

| **Supplementary Table 1. Communalities for the ORT-OUD items.** | |
| --- | --- |
| **Communalities** | |
| **Item** | **Extraction** |
| Family history of substance abuse [Alcohol] | 0.741 |
| Family history of substance abuse [Illegal drugs] | 0.694 |
| Family history of substance abuse [Prescription drugs] | 0.714 |
| Personal history of substance abuse [Alcohol] | 0.725 |
| Personal history of substance abuse [Illegal drugs] | 0.710 |
| Personal history of substance abuse [Prescription drugs] | 0.751 |
| Personal history of substance abuse [Age between 16-45 years] | 0.500 |
| Psychological disease [Attention deficit disorder (ADD), Obsessive compulsive disorder (OCD), Bipolar disorder, schizophrenia] | 0.757 |
| Psychological disease [Depression] | 0.546 |
| ***Abbreviations:*** *ADD: Attention deficit disorder; OCD: Obsessive compulsive disorder; ORT-OUD: Revised Opioid Risk Tool* | |

| **Supplementary Table 2. Correlation coefficients between the ORT-OUD total score and the ASSIST subscales scores.** | | | | |
| --- | --- | --- | --- | --- |
|  | **ORT-OUD** | | | |
|  | **General population (n=581)** | | **OUD patients (n=46)** | |
|  | **Correlation coefficient** | **p-value** | **Correlation coefficient** | **p-value** |
| **ASSIST-Opioids** | 0.174 | **<0.001** | 0.251 | 0.093 |
| **ASSIST-Sedatives** | 0.249 | **<0.001** | 0.598 | **<0.001** |
| **ASSIST-Alcohol** | 0.161 | **<0.001** | 0.100 | 0.510 |
| **Numbers in bold represent statistically significant values.*  *Abbreviations:* *ASSIST-Alcohol: Alcoholic beverages subscale of the Alcohol, Smoking, and Substance Involvement Screening Test; ASSIST-Opioid: Opioid subscale of the Alcohol, Smoking, and Substance Involvement Screening Test; ASSIST-Sedatives: Sedatives and sleeping pills subscale of the Alcohol, Smoking, and Substance Involvement Screening Test; ORT-OUD: Revised Opioid Risk Tool* | | | | |
